# Perioperative care of the geriatric patient for noncardiac surgery

**DOI:** 10.1002/clc.23302

**Published:** 2019-12-11

**Authors:** Jonathan D. Wolfe, Natasha K. Wolfe, Michael W. Rich

**Affiliations:** ^1^ Division of Cardiology Washington University School of Medicine St. Louis Missouri

**Keywords:** frailty, geriatric, postoperative complications, preoperative assessment, surgery

## Abstract

Adults age 65 and over are the fastest growing segment of the population in the United States and around the world. As the size of this population expands, the number of older adults referred for surgical procedures will continue to increase. Due to the physiologic changes of aging and the increased frequency of comorbidities, older adults are at increased risk for adverse outcomes, and perioperative care is inherently more complex than in younger individuals. In this review, we discuss the physiologic changes of aging relevant to the surgical patient, comprehensive preoperative assessment, and postoperative management of common complications in older adults in order to promote optimal clinical outcomes both perioperatively and long‐term.

## 1 INTRODUCTION

Adults age 65 and older are the fastest growing segment of the population in the United States (US) and around the world. As the population ages, the prevalence of chronic diseases such as hypertension, diabetes, heart failure, coronary artery disease, and Alzheimer's disease will substantially increase. These changes will result in increased demand for specialized care. In particular, the demand for surgical services such as general and vascular surgery is projected to increase by 18% and 31% respectively.^1^

Older adults often require a higher level of care in the perioperative setting compared to their younger counterparts. Physiologic changes of aging, comorbid conditions, and physical and cognitive impairment all predispose older patients to both cardiac and noncardiac perioperative complications as well as prolonged hospital stays. The American College of Surgeons National Surgical Quality Improvement Program (ACS NSQIP) and the American Geriatrics Society (AGS) have collaborated to provide Best Practice Guidelines regarding optimal perioperative assessment and management of geriatric patients.^2^, ^3^ These guidelines provide recommendations for the assessment and management of older surgical patients, incorporating current evidence, best practices, and expert opinion. This review will summarize the physiologic changes that occur with aging, discuss the role of preoperative risk assessment, and review the unique aspects of postoperative management in older adults.

## 2 PHYSIOLOGY OF AGING

Aging affects numerous biologic processes in every organ system. Changes associated with aging are inevitable, progressive, and result in increased susceptibility to disease. However, organ systems in a given person, and in different people, typically age at different rates depending on multiple factors including lifestyle, environment, and genetics. The end result is a continued decline in functional reserve, which impairs the body's ability to compensate for physiologic and pathologic stress. We will focus on some of the organ systems most relevant to perioperative care.

### 2.1 Cardiovascular system

Changes in the cardiovascular system related to aging are reviewed elsewhere in this issue of *Clinical Cardiology*. These changes are particularly relevant for intraoperative hemodynamic monitoring and perioperative care. Cardiovascular aging processes increase the risk for hypertension, coronary artery disease, valvular heart disease, heart failure, and arrhythmias. Taken together, these factors greatly increase the risk for perioperative cardiovascular complications in older adults, particularly type II myocardial infarction, acute heart failure, and atrial fibrillation.

### 2.2 Pulmonary system

The pulmonary system undergoes numerous age‐related changes that mirror many of the changes in the cardiovascular system. Aging results in a loss of pulmonary parenchyma and changes in supporting collagen fibers, resulting in decreased elastic recoil and decreased surface area for gas exchange. Approximately one‐third of the surface area per volume of lung tissue is lost over the course of a lifetime.^4^ Aging is associated with decreases in nearly all pulmonary function tests, including forced expiratory volume in 1 s and inspiratory and expiratory functional reserve.^5^

The chest wall becomes stiffer with age, significantly reducing chest wall compliance and increasing the work of breathing. This process is further aggravated as thoracic skeletal muscle is lost and the diaphragm flattens and becomes less efficient. Due to changes in closing capacity, which is the volume in the lungs at which the bronchioles collapse, combined with less efficient breathing mechanics, older adults can often fully expand their airways only in the standing position. This has implications for perioperative management as patients are often supine and intubated with resultant high rates of atelectasis. Cough is diminished due to decreased airway ciliary function and less efficient respiratory muscles. Pharyngeal sensation and the motor functions required to swallow also decrease. The combination of these anatomic and functional changes in the respiratory system increases the rate of postoperative pulmonary complications including aspiration, mucous plugging, pneumonia, and prolonged ventilator dependence.

### 2.3 Renal system

Aging is accompanied by a progressive loss of renal mass with the steepest decline after age 50. The greatest losses are seen in the renal cortex, affecting nephrons most important for urine concentration. Fat and fibrosis replace some of the remaining functional nephrons, and for individuals in their 70s, 10%‐30% of remaining nephrons are sclerotic, which further reduces the functional capacity of the renal system.^6^ The loss of nephrons is accompanied by a near 50% reduction in functioning glomeruli for individuals in their 70s compared to younger adults.

Creatinine clearance declines with age. However, due to loss of skeletal muscle mass and decreased creatinine production, serum creatinine may remain relatively stable despite substantial reductions in glomerular filtration rate. This makes estimation of glomerular filtration rate based on creatinine unreliable without an adjustment for age. At homeostasis, fluid and electrolyte balances are relatively maintained with aging. However, in times of stress, including the perioperative period, older kidneys have more difficulty maintaining circulating blood volume and managing sodium concentrations due to an inability to maximally dilute urine.^7^ Metabolic acidosis is also more common in older adults during the perioperative period related to a reduction in the kidneys' ability to acidify urine.

There is approximately a 10% reduction in renal blood flow per decade after age 50 related in part to intrarenal vascular changes. As a result, older kidneys have increased levels of vasodilatory prostaglandins and remain in a state of persistent vasodilation in order to compensate.^8^ This contributes to the roughly 2‐fold increase in risk of renal injury associated with the use of nonsteroidal anti‐inflammatory drugs in older adults. The loss of responsiveness of renal vascular tone also increases the vulnerability of the older kidney to ischemic insult from low cardiac output, hypotension, hypovolemia, and hemorrhage.

### 2.4 Neurologic system

While cardiopulmonary complications account for the largest portion of perioperative mortality, neurologic complications such as delirium are also very common. There is a gradual decrease in cortical gray matter starting in middle age. Age‐related neuron dropout is related to apoptosis, or programmed cell death, rather than injury, inflammation or another mechanism. Age‐related neuron loss is most prominent in the cerebellum and cortical gray matter. In later decades, there is a loss of the complexity of neuronal connections as white matter is lost and the dendritic tree of existing neuronal connections is pruned. The synthesis of neurotransmitters is also reduced, and combined with neuronal loss, processing speed and the ability of the older brain to integrate neural inputs can become more limited.^9^

Throughout life, neurons continue to form new synapses and new neurons are formed, but eventually the rate of loss exceeds the rate of gain.^10^ In some areas of the brain, dendritic connections increase with age, perhaps compensating for neuronal cellular loss. Certain memory functions, well‐practiced skills, and general knowledge remain stable or even slightly improve up until the seventh decade, at which point even these processes can decline.

The autonomic and peripheral nervous systems are also affected by age. There is neuronal loss in both sympathetic and parasympathetic ganglia combined with decreased adrenergic receptor responsiveness. As a result, there is a substantial increase in the concentration of circulating catecholamines and decreased responsiveness to exogenous catecholamines. In the peripheral nervous system, proprioceptors in the muscles, joints, and tendons diminish with age. Combined with decreased skeletal muscle innervation resulting in loss of motor units and decreased strength, coordination, and fine motor control, older adults are more prone to falls.

The combination of these changes may limit the ability of older patients to understand and process information in the perioperative period. Collectively, the changes in the central and peripheral nervous system increase the predisposition of older adults to numerous postoperative complications, including delirium, drug toxicity, and falls.

### 2.5 Pharmacokinetics and pharmacodynamics

Although older adults are the largest consumers of prescription drugs, they are often excluded from drug trials. This poses a challenge as aging leads to decreased lean body mass and increased body fat, which results in alterations in the distribution, clearance, and elimination of drugs. Furthermore, changes in cardiac output, age‐related increases in central nervous system sensitivity, and decreased renal or hepatic clearance of pharmaceuticals make older adults more prone to adverse drug events.

In summary, aging leads to a loss of physiologic reserve and reduced tolerance to physiologic and pathologic stress. Major surgery is highly stressful and invokes a surgical stress response with activation of the sympathetic nervous system and numerous hormonal pathways, as well as alterations in immune and hematologic function. Age‐associated declines in reserve capacity greatly diminish the older adult's ability to mount an effective stress response and increase the risk for perioperative complications, including death. In this context, frailty, a syndrome in which physiologic reserves are maximally invoked just to maintain day‐to‐day homeostasis, is strongly associated with increased postoperative mortality, complications, and prolonged hospital stays.

### 2.6 Perioperative assessment of the older adult

Perioperative assessment of the older adult poses challenges not encountered in younger patients. Changes in physiology due to aging and an increased prevalence of comorbid conditions place older adults at higher risk for major complications. Given their higher risk, it is important to elicit each patient's goals and priorities in the context of their overall health and likely surgical outcomes. In addition to a thorough cardiac evaluation, clinicians should also assess for conditions that are common in the older adult. The ACS NSQIP/AGS guidelines identify several areas of focus (Table 1), which we briefly summarize.

**Table 1:** Preoperative assessment checklist

Assessment	Clinical tools
• Screen for cognitive impairment and capacity	Mini‐cog assessment
• Screen for depression	Patient health questionnaire ‐ 2
• Screen for alcohol and other substance abuse/dependence	AUDIT‐C CAGE assessment
• Perform a preoperative cardiac evaluation	ACC/AHA algorithm
• Document functional status and history of falls; assess for frailty	Activities of daily living (ADLs) Independent activities of daily living (iADLs) Cumulative deficit model (Frailty Index)
• Take an accurate and comprehensive medication history; consider appropriate perioperative adjustments	Beers criteria
• Explore treatment goals and expectations	Code status
• Determine postoperative transition plan including family and social support	Healthcare power of attorney

### 2.7 Determine treatment goals and expectations

It is important to have discussions preoperatively regarding patient preferences and expectations from surgery as well as alternatives to surgery. Older patients are more likely to have multiple comorbidities and decreased functional status, which are associated with worse surgical outcomes.^11^ Many patients may forgo surgical treatment if it is likely to result in significant functional or cognitive impairment.^12^ Preferences regarding resuscitation and ventilator support should be clarified. Whenever possible, advance directives should be completed and included in the medical record. One study showed that 70% of individuals age 60 or older requiring end‐of‐life decisions lacked decision‐making capacity, increasing the importance of early conversations and advance directives.^13^ Furthermore, although clinicians often rely on family or other surrogates when patients are unable to make decisions, studies have shown that surrogates do not accurately represent patients' wishes in up to one‐third of cases.^14^ As part of these preoperative discussions, it is also important to determine a patient's family and social support systems.

### 2.8 Assessment of cognition, depression, and substance abuse

Cognitive impairment, dementia, depression, and substance abuse are causes of impaired sensorium and each is linked to postoperative complications. In particular, each of these conditions increases the risk of postoperative delirium, which is common in older adults undergoing major surgery and is associated with increased risk for falls, iatrogenic infections, and mortality, as well as longer hospital stays.^15^ Preoperative assessment of the older adult should include screening for potential causes of impaired sensorium so that appropriate preventative measures can be initiated and interventions can be implemented early in the postoperative period to minimize complications associated with these conditions.

Cognitive impairment and dementia are very common in older adults. The prevalence of dementia increases exponentially after the age of 65, affecting more than 13% of adults age 71 or older in the United States.^16^ The Mini‐Cog assessment is a simple tool that involves drawing a clock and recalling three items at 3 min that is helpful in screening for cognitive impairment. Individuals that screen positive may undergo a more in‐depth evaluation.

Depression is common among older adults and has been linked to increased mortality and longer postoperative stays after surgery.^17^ Depression is also associated with higher pain perception and increased requirement for postoperative analgesic use^.17^ There are numerous screening tools for depression that can be used to identify patients at risk. The two‐question patient health questionaire‐2 (PHQ‐2) has a 97% sensitivity and 67% specificity for identifying depression in older adults. A positive result on a screening questionnaire should prompt clinicians to further explore the likelihood of depression or refer patients to an appropriate provider for additional evaluation and treatment.

The majority of older adults in the US consume alcohol. While most older adults drink in moderation, at‐risk behavior is common. Alcohol abuse and dependence increase the risk for postoperative mortality and other complications including iatrogenic infections and prolonged length of stay.^3^ If at‐risk or hazardous drinking behavior is identified preoperatively, motivated patients may be referred to a substance abuse program and clinicians may consider preoperative pharmacologic strategies to prevent relapse. For patients with alcohol dependence for whom an operation cannot be delayed, perioperative treatment with multivitamins and high dose thiamine is prudent.^3^

For individuals with altered sensorium or impaired cognition, it is important to assess decision‐making capacity as it relates to the ability to provide informed surgical consent. Clinicians should assess the four legal criteria for decision‐making capacity, which include the ability to clearly indicate treatment choice; understand the relevant information communicated by the healthcare team; acknowledge his or her medical condition, treatment options, and likely outcomes; and engage in a rational discussion about treatment options. For individuals deemed not to have capacity, a surrogate decision maker should be identified and the risks and benefits of proceeding with surgery should be reevaluated.

### 2.9 Assessment of functional status, fall risk, and frailty

Functional status is one of the strongest predictors of postoperative mortality.^3^ In addition to determining metabolic equivalents (METS) as part of the cardiac evaluation, evaluating baseline functional status in the older adult includes assessment of whether basic and instrumental activities of daily living (ADLs and IADLs) can be performed independently, including getting out of bed, dressing, bathing, meal preparation, and shopping. Obtaining a fall history is also important, as falls are the primary cause of unintentional injury in older adults.^18^ The Timed Up and Go Test is useful to assess for increased risk of falls. This simple test can be performed in the office and consists of asking the patient to rise from the chair, walk 10 ft, return to the chair, and sit down again. Individuals that require more than 15 s to complete the test are at high risk of falls and may benefit from a preoperative referral to physical therapy.

Frailty is a syndrome of decreased physiologic reserve and diminished capacity to adapt to physiologic and pathologic stressors. In numerous studies, frailty has been linked to poor health outcomes, including functional decline, falls, increased number of hospitalizations, and death. Once recognized, clinicians may attempt interventions to reduce frailty, including strengthening exercises, physical therapy programs, Tai Chi, protein supplementation, and vitamin D supplementation, but the value of these interventions for improving clinical outcomes in patients with frailty is controversial.^19^

### 2.10 Perform a perioperative cardiac and pulmonary evaluation

**Figure 1:**
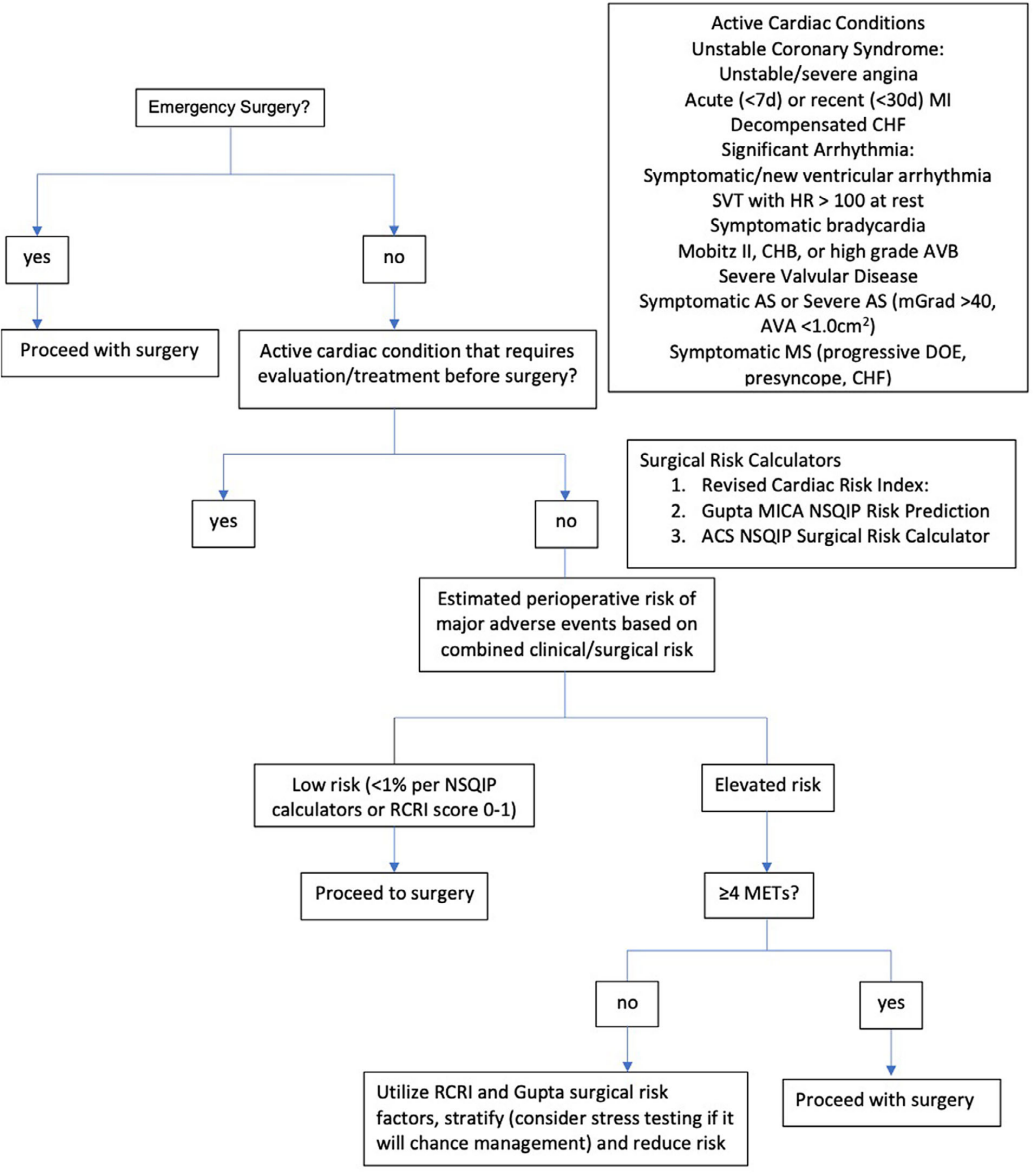
Preoperative Cardiac Risk Assessment Algorithm. MI, myocardial infarction; CHF, congestive heart failure; SVT, supraventricular tachycardia; CHB, complete heart block; AVB, atrioventricular block; AS, aortic stenosis; MS, mitral stenosis; mGrad, mean gradient; AVA, aortic valve area; DOE, dyspnea on exertion; MICA, myocardial infarction or cardiac arrest; NSQIP, National Surgical Quality Improvement Program; ACS, American College of Surgeons; RCRI, revised cardiac risk index; METs, metabolic equivalents. Adapted from Peri‐operative cardiovascular evaluation for noncardiac surgery (2014) ACC/AHA, J Am Coll Cardiol. 2014;64(22):e94

Older adults are at significantly higher risk for perioperative cardiac adverse events than their younger counterparts. A thorough preoperative cardiac evaluation should be performed in accordance with the American College of Cardiology (ACC) / American Heart Association (AHA) Guideline and should include consideration of the type and urgency of the surgical procedure, assessment of the patient's functional capacity, and optimization of the individual's cardiac risk profile (Figure 1).^20^ Patients' perioperative risk of major adverse cardiac events (MACE) may be calculated and can be considered low (<1%) or high (>1%). As previously discussed, decreased functional capacity is associated with higher postoperative mortality and an increased risk of cardiac complications. If a patient is able to perform ≥4 METS (climbing a flight of stairs, walking on level ground at 4 mph, performing heavy housework) then he or she is considered to have a moderate or greater functional capacity and no further testing is indicated in the absence of active cardiac ischemia, heart failure, or uncontrolled arrhythmia. If the patient's baseline functional capacity is unclear or cannot be easily assessed, it should be presumed to be low when evaluating perioperative risk. Patients with active cardiac conditions should be managed in accordance with ACC/AHA guidelines. These guidelines recommend using preoperative cardiac testing judiciously, and ordering tests only if the results will change management and after discussion with patients using a shared‐decision making process.

Postoperative pulmonary complications are common following noncardiac surgery and contribute significantly to mortality and morbidity in older adults. Recent reviews show that postoperative pulmonary complications have a similar prevalence to cardiac complications.^5^ Risk factors for pulmonary complications include pre‐existing pulmonary disease, obstructive sleep apnea, and current cigarette use. Strategies to prevent perioperative pulmonary complications include preoperative optimization of symptoms for patients with existing lung disease and smoking cessation.

### 2.11 Take an accurate medication history and adjust for polypharmacy

It is imperative to obtain a complete list of a patient's medications prior to surgery, including over‐the‐counter medications, vitamins, and herbal supplements. Medications may be classified as essential and nonessential. Nonessential medications that increase surgical risk or medications that pose a high risk for serious drug‐drug interactions should be discontinued if appropriate. Herbal medications in particular should be stopped at least a week prior to elective surgery due to their uncertain contents. Essential medications that should be continued perioperatively include those with withdrawal potential such as selective serotonin reuptake inhibitors, tricyclic antidepressants, and beta‐blockers. New prescriptions for benzodiazepines, zolpidem, and medications with anticholinergic side effects, such as antihistamines, should be avoided as these can increase the risk of postoperative delirium and falls.

## 3 POSTOPERATIVE MANAGEMENT OF THE OLDER ADULT

Older patients are at an increased risk for postoperative complications including delirium, falls, poor nutrition, urinary tract infections (UTIs) and other iatrogenic infections, pressure ulcers, and functional decline.^2^ In addition to these more geriatric‐specific complications, older adults have higher rates of cardiac and pulmonary complications, venous thromboembolic disease (VTED), and acute kidney injury (AKI). Table 2 provides an overview of these complications and their management, which may be applied where appropriate.

**Table 2:** Postoperative rounding considerations

Daily Evaluation	Screening measures	Risk factors	Prevention/management strategies
• Delirium/cognitive impairment	Confusion assessment method (CAM) short form and ICU form	Cognitive impairment/dementia Poor vision or hearing Infection Critical illness	Optimize physical environment (sleep hygiene, minimize restraints, family at bedside) Vision and hearing aids accessible Avoid potentially inappropriate medications (Beers criteria) Minimize use of psychoactive medications
• Acute pain	Clinical Assessment	Chronic use of alcohol Depression	Individualized preoperative pain management plan Opioid‐sparing techniques when possible Bowel regimen when opioids are used Vigilant dose titration
• Fall risk	Morse Fall Scale	Altered mental status Dehydration History of falls Impaired mobility Visual impairment	Universal fall precautions Early mobilization Early PT/OT Assistive walking devices Scheduled toileting
• Nutritional status	Dietary consultation Swallowing assessment	Cognitive impairment/dementia Frailty Recent orthopedic procedures	Resume diet as early as able Dentures available Supplementation if indicated
• UTI prevention	Clinical Assessment	Indwelling catheter use, urinary retention	Daily documentation of Foley catheter indication Catheter care bundles, hand hygiene, barrier protection
• Pressure Ulcers	Braden scale Norton risk‐assessment	Contracture Edema Incontinence Limited mobility Loss of sensation	Reduce pressure, friction, humidity Maintain adequate nutrition Frequent repositioning and early mobilization Wound care
• Pulmonary complications	Clinical Assessment	Chronic lung disease Aspiration	Chest physiotherapy and incentive spirometry Early mobilization Aspiration precautions
• VTE complications	Clinical Assessment	Immobility Active cancer Orthopedic surgery	Prophylactic subcutaneous heparin or enoxaparin Early ambulation Mechanical compression devices
• Functional decline	Clinical Assessment	Frailty Cognitive impairment/dementia Depression Poor mobility Comorbid conditions	Multidisciplinary rounds Early mobilization and PT/OT Family participations Nutritional support Minimize patient tethers

*Note*: Adapted from Optimal perioperative management of the geriatric patient: a best practices guideline from the American College of Surgeons NSQIP and the American Geriatrics Society. *J Am Coll Surg* 2016; 222 (5):930–47.

### 3.1 Delirium and cognitive impairment

Delirium is an acute decline in cognitive function and attention. It is classically described as waxing and waning, with periods of lucidity followed by periods of altered consciousness. Delirium is the most common postoperative complication in older patients and occurs in 14%‐56% of patients with the greatest incidence after high risk surgeries; the prevalence may exceed 80% for patients requiring mechanical ventilation in an intensive care unit setting.^2^, ^20^, ^21^ Delirium is associated with worse surgical outcomes, longer hospital stays, functional decline, long‐term cognitive impairment, institutionalization, and increased mortality. 2, 22, 23, 24

Fortunately, an estimated 30%‐40% of delirium cases are considered preventable.^2^, ^23^ There are several nonpharmacologic interventions that can prevent the development of delirium, including early mobility, maintenance of normal sleep/wake cycles, use of assistive devices (glasses and hearing aids), a calm environment with family and familiar objects in the room, adequate pain management, prevention of constipation, avoidance of deliriogenic medications, and minimal use of patient restraints. If a patient does develop delirium, clinicians should look for and treat possible precipitating conditions including uncontrolled pain, hypoxia, infections, urinary retention, fecal impaction, electrolyte abnormalities, and hypoglycemia. In addition, a careful review of all medications administered in the hospital (including PRNs) should be performed and all deliriogenic medications should be stopped. 23

Many medications can induce delirium in older adults and should be avoided in the postoperative setting (Table 3). Anticholinergics, sedative‐hypnotics, corticosteroids, and meperidine have been associated with increased risk of delirium.^20^ Adequate pain control should be a priority, but nonopioid options should be utilized when possible. The AGS recently released updated Beers Criteria for potentially inappropriate medications in older adults.^25^ Careful consideration of the risks and benefits of each medicine should be performed to customize therapy for individual patients.

**Table 3:** Common deliriogenic medications

Category	Examples	Strength of recommendation	Quality of evidence
Anticholinergics	First generation antihistamines: chlorpheniramine, diphenhydramine, doxylamine, hydroxyzine, meclizine, promethazine Antispasmotics: atropine, hyoscyamine, scopolamine Antidepressants: amitriptyline, nortriptyline, paroxetine	Strong	Moderate
Antipsychotics^a^	First generation: chlorpromazine, fluphenazine, haloperidol, thioridazine Second generation: aripiprazole, clozapine, olanzapine, quetiapine, risperidone	Strong	Moderate
Benzodiazepines	Short acting: alprazolam, lorazepam, temazepam Long acting: chlordiazepoxide, clonazepam, diazepam	Strong	Moderate
Corticosteroids^b^		Strong	Moderate
H2‐receptor antagonists	Cimetidine, famotidine, ranitidine	Strong	Weak
Meperidine	—	Strong	Moderate
Nonbenzodiazepine, benzodiazepine receptor agonists	Eszopiclone, zaleplon, zolpidem	Strong	Moderate

*Note*: Adapted from the American Geriatrics Society 2019 updated AGS Beers criteria for potentially inappropriate medication use in older adults. *J Am Geriatr Soc*. 2019;67(4):674‐94.

a May be required to treat concurrent mental health conditions but should be prescribed at the lowest effective dose and for the shortest possible duration.

b Excludes inhaled and topical forms. Oral and parenteral steroids may be required for some conditions such as chronic obstructive pulmonary disease but should be prescribed at the lowest effective dose and for the shortest possible duration.

Daily screening for delirium by a trained healthcare professional with the use of validated tools, such as the Confusion Assessment Method, should be considered, especially in high‐risk patients.^26^ However, evidence to support daily screening for delirium is inconclusive, and no randomized controlled trial has been completed on this topic. When delirium is suspected, the diagnosis can be made using the Diagnostic and Statistical Manual criteria or the CAM Algorithm.^27^ Once diagnosed, treatment of delirium should primarily focus on nonpharmacologic interventions, which mirror the interventions for prevention of delirium that were previously discussed.

Pharmacologic therapy for the management of delirium should be considered only after attempts with nonpharmacologic interventions have been unsuccessful. Best practice guidelines from the AGS recommend that low dose antipsychotics for the shortest duration possible can be employed only in the setting of agitated or distressed patients that pose harm to themselves or others.^23^

### 3.2 Falls

Falls are estimated to occur in approximately 30% of community‐dwelling adults age 65 or over every year.^28^ Older adults in hospitals or nursing homes fall significantly more often than their community‐dwelling counterparts. Falls result in injury about one third of the time, with serious injuries such as bone fractures or head injuries in 5%.^29^ It is therefore important to conduct a risk assessment for falls in older adults and to use interventions aimed at reducing fall risk. Risk factors for falls include altered mental status, frequent toileting, dehydration, history of falls, impaired mobility, sedating medications, and visual impairments.^30^ The Agency for Healthcare Research and Quality developed a fall prevention hospital toolkit outlining Universal Fall Precautions that hospitals can implement to minimize falls.^31^ These precautions include familiarizing patients with the hospital environment, maintaining the call light and personal possessions within reach, nonslip footwear, night lights, and keeping patient care areas uncluttered. If specific risk factors for falls are noted, targeted interventions should be implemented to reduce fall risk. Importantly, fall prevention strategies should minimize the use of restraints and should not deter mobilization and ambulation postoperatively.

### 3.3 Nutrition

Nearly 40% of hospitalized older adults are considered malnourished.^32^ Malnutrition increases the risk of adverse outcomes in hospitalized patients, including mortality, readmissions, and increased length of stay.^33^ For these reasons, it is recommended that older postoperative patients be evaluated for adequate nutritional intake and for aspiration risk on a daily basis. Signs of aspiration include cough or choking with swallowing, drooling, and changes in voice or speech. Patients who use dentures should have them readily available. It is also recommended that aspiration precautions be utilized, including elevating the head of the bed and sitting upright while eating. Oral nutritional supplementation is recommended in older hospitalized patients to treat malnutrition and for those at risk of developing malnutrition, including frail patients, those with dementia, and patients following orthopedic surgery.^2^

### 3.4 Urinary tract infections

Older patients are at increased risk of iatrogenic infections postoperatively, particularly urinary tract infections (UTI), which account for up to 40% of all iatrogenic infections.^34^ Close attention should be paid to prevent the development of UTIs, including appropriate indications/use of indwelling catheters, sterile insertion techniques, good hand hygiene, and avoidance of routine catheter irrigation. If a patient has an indwelling catheter, review of its ongoing need should be done on a daily basis, and the catheter should be removed as soon as appropriate. Notably, indwelling catheter use is not generally indicated for incontinent patients.^2^

### 3.5 Pressure ulcers

With aging, adults lose subcutaneous tissue and develop decreased skin elasticity. If older surgical patients are immobilized, they are at high risk for the development of pressure sores. It is estimated that up to two‐thirds of pressure sores occur in older hospitalized adults. Pressure ulcers can lead to secondary infections and prolonged hospital stays. Risk factors for pressure ulcers include advanced age, abnormal positioning due to contracture, edema, chronic moisture, incontinence, limited mobility, and loss of sensation. After identifying patients at risk, preventive measures should be undertaken, including repositioning patients every 1‐2 hours, using low‐pressure air or air‐fluidized mattresses, ensuring adequate nutrition, and daily skin inspection.^2^ Treatment of pressure ulcers should include good wound care, debridement of nonviable and devascularized necrotic tissue, and consideration of waste management systems in patients who are incontinent.

### 3.6 Other acute postoperative complications

Older adults are also at increased risk of cardiac, pulmonary, thromboembolic, and renal complications postoperatively. History of heart failure is a significant predictor of perioperative cardiovascular risk. If patients have decompensated heart failure preoperatively, postoperative risk for mortality is high. Thus, patients with decompensated heart failure should be treated aggressively and surgery should be delayed if feasible until volume and hemodynamic status have been optimized. Postoperative atrial fibrillation (AF) is common and increasing age is the strongest risk factor for its development.^35^ Management of hemodynamically stable patients with AF includes correction of precipitating factors (hypoxia, inadequate pain control, electrolyte imbalances, etc.), rate control with AV‐nodal blocking agents, and anticoagulation (unless contraindicated) to prevent thrombus formation. Postoperative AF generally converts to sinus rhythm as patients recover from the early postoperative hyper‐adrenergic and pro‐inflammatory state. Thus, efforts to achieve rhythm control via direct electrical cardioversion or with antiarrhythmic agents are generally not recommended unless the patient is highly symptomatic, hemodynamically unstable, or remains in AF >24‐48 hours.^36^ Elevation of cardiac biomarkers such as troponin following surgery is common and in most cases can be attributed to supply/demand imbalance or an entity called troponin elevation after noncardiac surgery rather than to acute coronary syndrome and can be managed conservatively.

Due to physiologic changes of aging, older adults are risk for postoperative pulmonary complications such as atelectasis, hospital‐acquired pneumonia, and respiratory failure. Close attention should be paid to optimize respiratory status postoperatively with aspiration precautions, use of incentive spirometry, and early mobilization.^2^ With advancing age, the risk of thrombosis increases. In the postoperative setting, older patients are at higher risk of VTE compared to younger cohorts. Prophylaxis with subcutaneous heparin or enoxaparin should be implemented unless contraindicated. In high‐risk settings (malignancy, orthopedic surgery, trauma), VTE may occur in up to 50% of cases if prophylaxis is not given.^36^ Early ambulation and mechanical compression devices can also be used to prevent development of VTE. Older patients are at higher risk for development of acute kidney injury due to decreased renal reserve and hemodynamic changes associated with surgery. Risk can be minimized by avoiding nephrotoxic drugs and optimizing volume status. Older patients have reduced capacity to regulate body temperature and are therefore at increased risk for developing hypothermia, especially those with low weight and/or frailty. In turn, hypothermia is associated with increased risk for infections, bleeding, cardiac arrhythmias, electrolyte abnormalities, altered drug metabolism, and prolonged length of stay, as well as patient discomfort. In most cases, hypothermia is mild and can be treated with warming blankets.

### 3.7 Functional decline and long‐term complications

Hospitalizations for surgery or medical illness place older adults at increased risk for functional decline, not only during the hospitalization but also during long‐term follow‐up. More than 30% of older hospitalized adults develop a new disability that impairs their ADLs. Less than half of these patients recover to prior functional status at 1 year post hospitalization.^37^ Many of the risk factors for falls are also risk factors for functional decline during hospitalization, including advanced age, frailty, cognitive impairment, poor mobility, depression, and comorbid conditions. Preventive efforts should be undertaken to minimize the risk of functional decline postoperatively. Interventions to prevent functional decline include early mobilization, early physical and occupational therapy evaluation, nutritional support, promotion of family participation in recovery, and comprehensive discharge planning.^38^ Novel programs of enhanced medical rehabilitation have also been shown to improve functional outcomes in high‐risk older patients discharged to postacute care facilities.^39^

### 3.8 Postoperative care planning

Ensuring smooth care transitions between health care settings is critical to the care of older patients, and discharge planning should ideally begin prior to surgery. The transfer of care from hospital to community settings can pose challenges that can place patients at higher risk for emergency room visits, readmission, and functional decline. Clear communication between inpatient health care providers, patients, family/caregivers, and primary care providers is of the utmost importance. The inpatient team should assess patients' social support and need for postacute care, including inpatient rehabilitation, skilled nursing facilities, or home health. Family and caregivers should be included in discharge planning. Prior to discharge, older adults should undergo assessment of nutrition, cognition, ambulation, functional status, and the presence of delirium. Appropriate follow‐up for all identified conditions should be arranged.^2^

## 4 CONCLUSIONS

The preoperative evaluation of older patients requires not only an assessment of cardiovascular risk, but also screening for cognitive impairment and dementia, depression, substance abuse, fall risk, functional deficits, and frailty. A careful preoperative medication assessment with minimization of nonessential and inappropriate medicines can prevent complications from polypharmacy. Postoperatively, evaluation and preventative interventions for common geriatric complications such as delirium, falls, malnutrition, pressure ulcers, and UTIs should be performed on a routine basis. Older surgical patients are not only at increased risk for short‐term perioperative complications, but these complications also increase the risk for long‐term adverse outcomes, including cognitive impairment, functional decline, and diminished quality of life. Ensuring smooth postoperative transitions of care is of critical importance and can help prevent readmissions and functional decline.

## CONFLICT OF INTEREST

We have no conflicts of interest to disclose.
